# Unveiling Switching Function of Amino Acids in Proteins Using a Machine Learning Approach

**DOI:** 10.1021/acs.jctc.3c00665

**Published:** 2023-11-07

**Authors:** Parisa Mollaei, Amir Barati Farimani

**Affiliations:** † Department of Mechanical Engineering, Carnegie Mellon University, 5000 Forbes Avenue, Pittsburgh, Pennsylvania 15213, United States; ‡ Department of Biomedical Engineering, Carnegie Mellon University, 5000 Forbes Avenue, Pittsburgh, Pennsylvania 15213, United States; ¶ Machine Learning Department, Carnegie Mellon University, 5000 Forbes Avenue, Pittsburgh, Pennsylvania 15213, United States

## Abstract

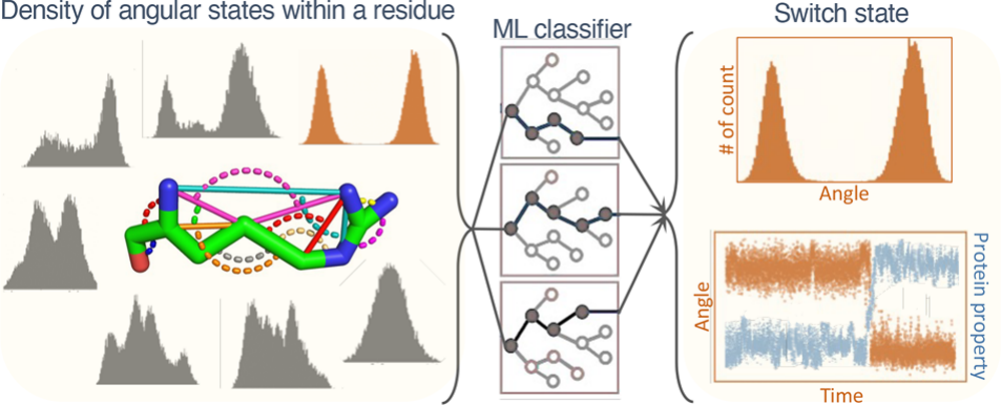

Dynamics of individual amino acids play key roles in the overall properties of proteins. However, the knowledge of protein structural features at the residue level is limited due to the current resolutions of experimental and computational techniques. To address this issue, we designed a novel machine-learning (ML) framework that uses Molecular Dynamics (MD) trajectories to identify the major conformational states of individual amino acids, classify amino acids switching between two distinct modes, and evaluate their degree of dynamic stability. The Random Forest model achieved 96.94% classification accuracy in identifying switch residues within proteins. Additionally, our framework distinguishes between the stable switch (SS) residues, which remain stable in one angular state and jump once to another state during protein dynamics, and unstable switch (US) residues, which constantly fluctuate between the two angular states. This study also illustrates the correlation between the dynamics of SS residues and the protein’s global properties.

## Introduction

A protein is comprised of amino acids, and their dynamics play crucial roles in determining the characteristics of the protein.^1−23^ However, investigating the structures and dynamics of amino acid residues within a protein poses challenges both experimentally and computationally.^4,6,24−28^ Although techniques such as X-ray crystallography and NMR spectroscopy can be used to characterize the structure and dynamics of amino acids,^11,29,30^ current spatiotemporal experimental resolutions prohibit the accurate descriptions of their dynamical features.^12,31−35^ The rapid motion of amino acids occurs at frequencies in the gigahertz (GHz) range, which cannot be precisely resolved by current experimental instrumentation. However, investigating conformational changes of amino acids with femtosecond (fs) resolution is practical using molecular dynamics (MD) simulations,^36−41^ which is valuable data for obtaining information about the 3D conformational dynamics of proteins.^36,40,41^ In the case of individual amino acids within a protein, one notable challenge arises from rare events such as switching to different conformations. These events may not be observed in long trajectory simulations. Indeed, utilizing numerous short trajectories generated from various conformational states of proteins enables us to statistically capture those rare events. However, analyzing this huge amount of data poses another challenge due to the high dimensionality of the data. Given these problems with MD simulation sampling, we addressed a couple of questions in this study: 1. Can we identify major conformational states of single amino acid residue using sufficient MD trajectories and classify those switching between two distinct structures? 2. Can we evaluate the degree of dynamic stability of the switch residues (or if the two conformations are stable?)? 3. Which of the switch residues contributes to the overall function of the protein? To answer these questions, knowledge of statistics and machine learning (ML) is required to deal with the large amounts of the data.^42−46^ ML models can automatically uncover patterns and relationships in the data, which can be used to infer and predict physical phenomena.^43,47,48^ In addition, ML can be used to take advantage of thousands of short trajectories.^49−52^ Inspired by the aforementioned challenges and the power of ML, we developed a framework that processes MD trajectories and uses ML models to characterize amino acid conformations and dynamics. Aggregation of amino acid residue dynamics at different conformational states and extracting biophysical knowledge from short trajectories can be viable by projecting residue structural features onto the Density of States (DoS). For the structural features of an amino acid, we focused on the angles formed by the three atoms. If a residue experiences no significant conformational changes, it leads to a unimodal density of angular states. On the other hand, some amino acid residues undergo transitions between different conformations resulting in shifts between two or more angular states. For the purpose of this investigation, we defined residues that demonstrate bimodal DoS with no intermediate state(s) as switch residues. The proposed approach can be developed to investigate the switching function of residues up to n-modal where all the modes may play critical roles in protein dynamics and function. However, in this study, we focused on bimodal switch residues and aimed to introduce a new metric to analyze dynamics of a given protein at the residue level. The ultimate objective of our study is to investigate potential correlations between the functionality of switch residues and the overall properties of proteins.

## Methods

**Figure 1:**
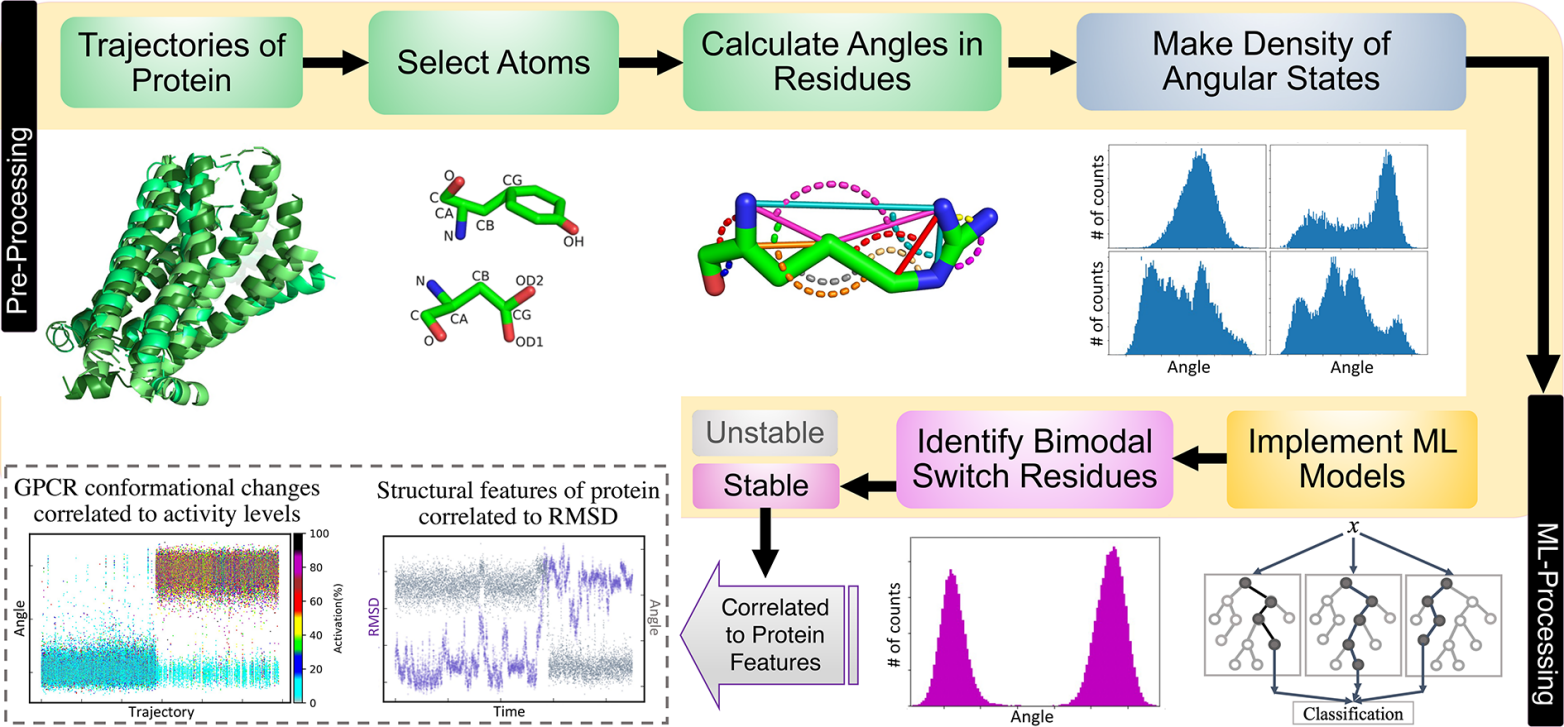
Framework for classifying amino acid residues into switch and nonswitch residues in proteins using MD simulations and ML models. The framework consists of preprocessing of protein trajectories and ML-processing. It is followed by two examples of using this method in Fs peptide protein and the β_2_*AR* receptor.

Our framework processes the MD trajectories of any protein and iterates over all of the residues within it. Figure 1 describes this framework that characterizes the switching function of residues in proteins. It consists of two main sections: preprocessing and ML-processing. In the preprocessing section, we calculate all of the angles within every single residue along the trajectories and build the histogram (DoS) for each of the angles. For the ML-processing section, we prepared a training data set for ML models consisting of labels and features of the DoS to classify individual residues into switch or nonswitch. Finally, to validate the performance and effectiveness of our method, this framework is applied to trajectory data sets of two different proteins: Fs peptide protein and β_2_*AR* receptor.

### Data Preprocessing

To extract information from the simulations, we considered residues as rigid bodies and identified only atoms located at the nodes and edges of their structures (see Table S1 for a list of selected atoms in amino acids). The whole structure of amino acid, along with its dynamics, can be effectively described through all the angles formed by combinations of three of these atoms. The reason we chose the specific subset of atoms is to decrease the large number of possible angles within a single amino acid. For instance, 12 atoms within TYR residue (N, CA, CB, CG, CD1, CE1, CZ, OH, CE2, CD2, C, O) result in a total of 220 angles, however, by implementing the atom selection approach, we were able to significantly reduce them to 35 angles created by seven atoms (N, CA, CB, CG, OH, C, O). Using the selected atoms, we first measured each angle for every frame of trajectories in order to provide a DoS for the angle. Generally, angles formed by sets of three atoms provide more detailed information about the local spatial arrangement of atoms surrounding a central atom and dynamics of an amino acid compared to dihedral angles (Supporting Information, Section 2). Therefore, for large proteins, notable challenges arise due to both the size of the data throughout the entire protein^53^ and extremely diverse patterns of DoS within it. To overcome these challenges, we employed ML models to accurately classify the switch residues.

### Training ML Models

**Figure 2:**
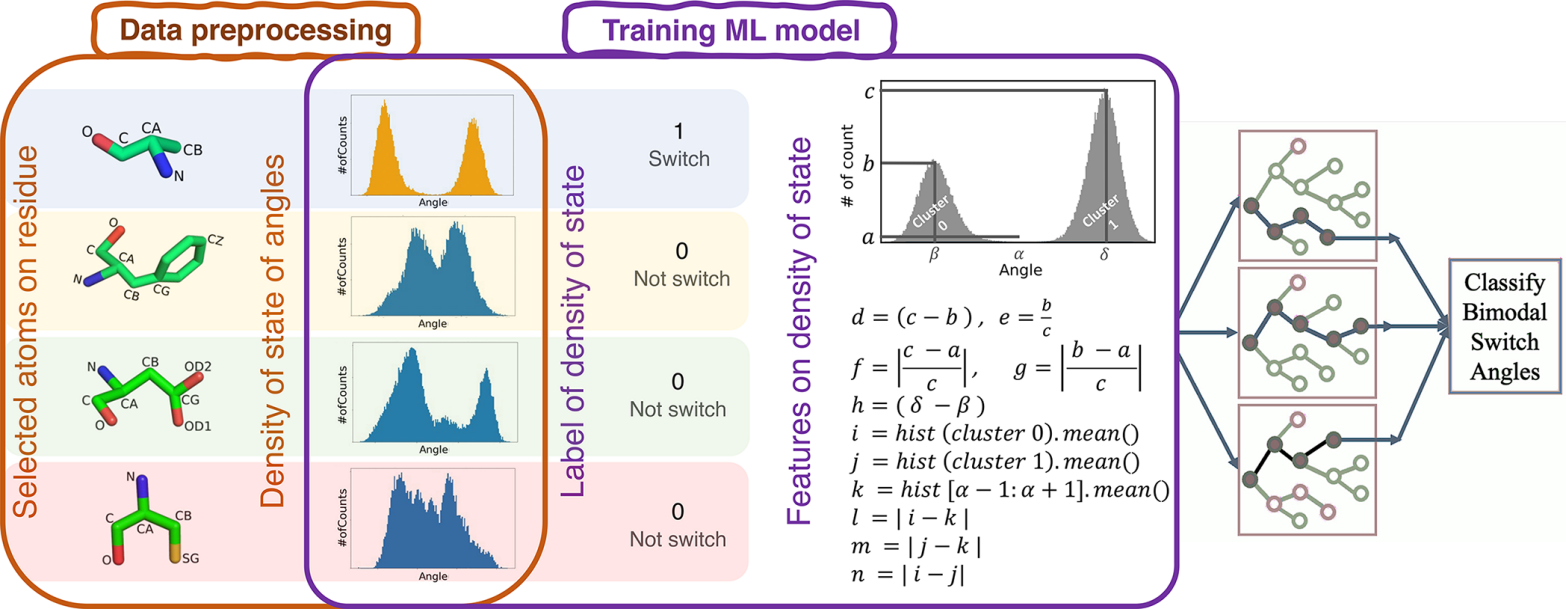
Data preprocessing and training ML models process to classify switch and nonswitch residues.

To train ML models capable of classifying switch residues within a protein, a data set containing information on angular states as well as their labels is required. After obtaining various patterns of distributions of the DoS, we labeled them by visual inspection, determining whether a given density represents a switch or not. As shown in Figure 2, the labels for bimodal DoS with no state(s) in between are 1 and others are 0. Besides the labels, the features of the DoS are required for training ML models. We found that the raw data of DoS will yield low accuracy if used as features. Therefore, we defined and engineered 14 features based on patterns of distributions of the bimodal switch histograms as input for the ML models (Figure 2). We trained three ML models: the Decision Tree model which builds a tree-like structure by recursively splitting data based on the features’ values, the Random Forest model which creates an ensemble of multiple decision trees and combines their predictions through voting or averaging,^54^ and the XGBoost model that uses a gradient boosting framework to build an ensemble of learners (usually decision trees).^55^ We benchmarked the accuracy of different ML algorithms and found that the Random Forest model demonstrated superior test accuracy of 96.94% compared to the XGBoost and Decision Tree models (Table 1). To obtain this accuracy, we used 5-fold cross-validation.

**Table 1:** Performance of Random Forest, XGBoost, and Decision Tree Models in Classifying Switch and Nonswitch Residues^a^

ML Model	Switch classification accuracy (%)
Random Forest	96.94 (0.04)
XGBoost	96.42 (0.03)
Decision Tree	95.91 (0.03)

a The standard deviations are shown in parentheses.

## Characterizing Stability

**Figure 3:**
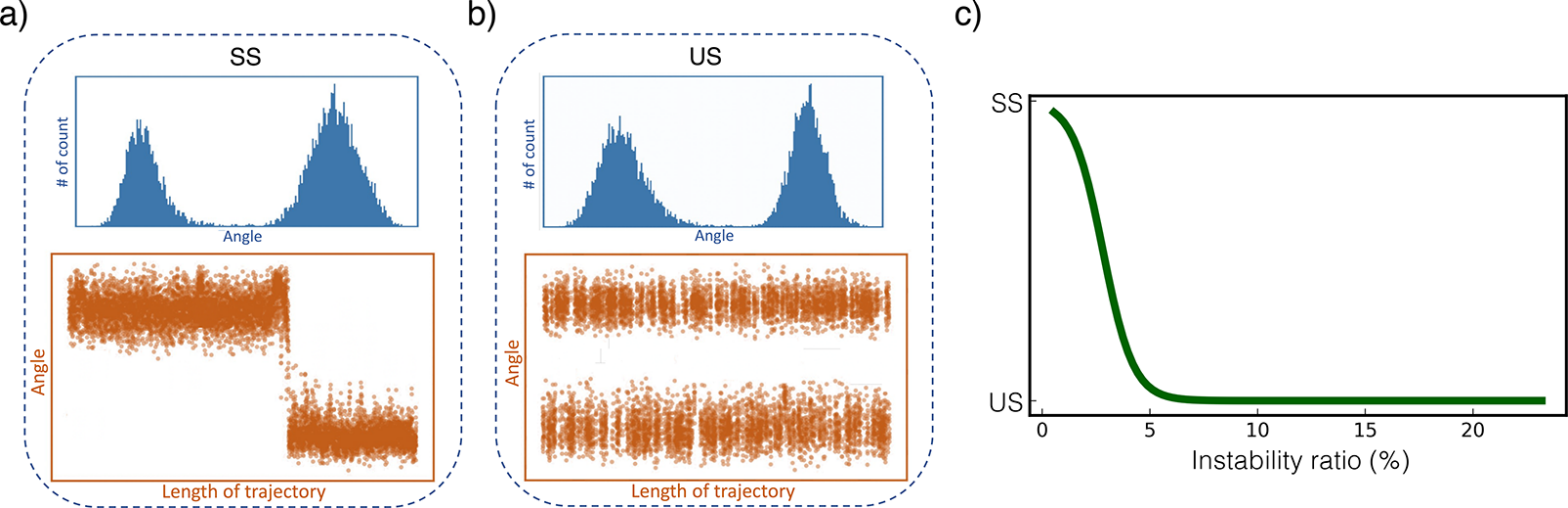
Comparative representation of switch residues exhibiting a similar DoS but different dynamics. DoS along with dynamical representation for (a) stable switch (SS) residue and (b) unstable switch (US) residue in a protein. (c) Established Instability ratios by the Logistic Regression model to perform classification of switch residues into SS and US residues.

While the switch residues exhibit similar DoS patterns (blue histograms in Figure 3a,b) we observed that their kinetics (dynamics) may be totally different. The brown plot in Figure 3a shows that the conformation of the switch residue changes with a single transition between the two angular states during the protein dynamic. On the other hand, another switch residue (shown in the brown plot in Figure 3b) constantly oscillates between the two angular states throughout the same trajectory. It can be inferred that the DoS can accurately determine the switching modes within a residue, but it lacks the ability to identify the dynamic stability of the switches. To address this challenge, we developed another framework aimed at classifying the two categories of switch residues into stable switch (SS) (Figure 3a) and unstable switch (US) (Figure 3b) classes. To differentiate between the SS and US residues, we defined an Instability ratio which is characterized as the ratio of total transitions between the two angular states over the length of the trajectories (%). To determine the total transitions between the two angular states, we need to know which point belongs to which of the angular states. To obtain this information, we used clustering techniques in an unsupervised fashion. We utilized the k-means algorithm with two clusters using the Scikit-learn library.^56^ It was observed that the Instability ratio effectively differentiates the SS and US residues when it is either below 1% (Figure S2a) or above 6% (Figure S2b). However, it becomes challenging to distinguish between the two in cases where the Instability ratio falls within the intermediate range (1% < Instability ratio < 6%) (Figure S2c,d). The reason for this lies in the diverse distribution of transitions’ timesteps between the two angular states throughout the trajectories (see Supporting Information Section 3). To resolve this challenge, we trained the Logistic Regression model with Instability ratio to establish the appropriate Instability ratio for classifying SS and US residues. The training data set for the model is similar to the brown plots in Figure 3 containing the Instability ratio and labels (SS or US). Ultimately, the model achieved an accuracy of 98.97% with a standard deviation of 0.01 for classifying the SS and US residues using 5-fold cross-validation. Figure 3c illustrates how the Logistic Regression model established the Instability ratio to classify the SS and US residues.

## Results and Discussion

**Figure 4:**
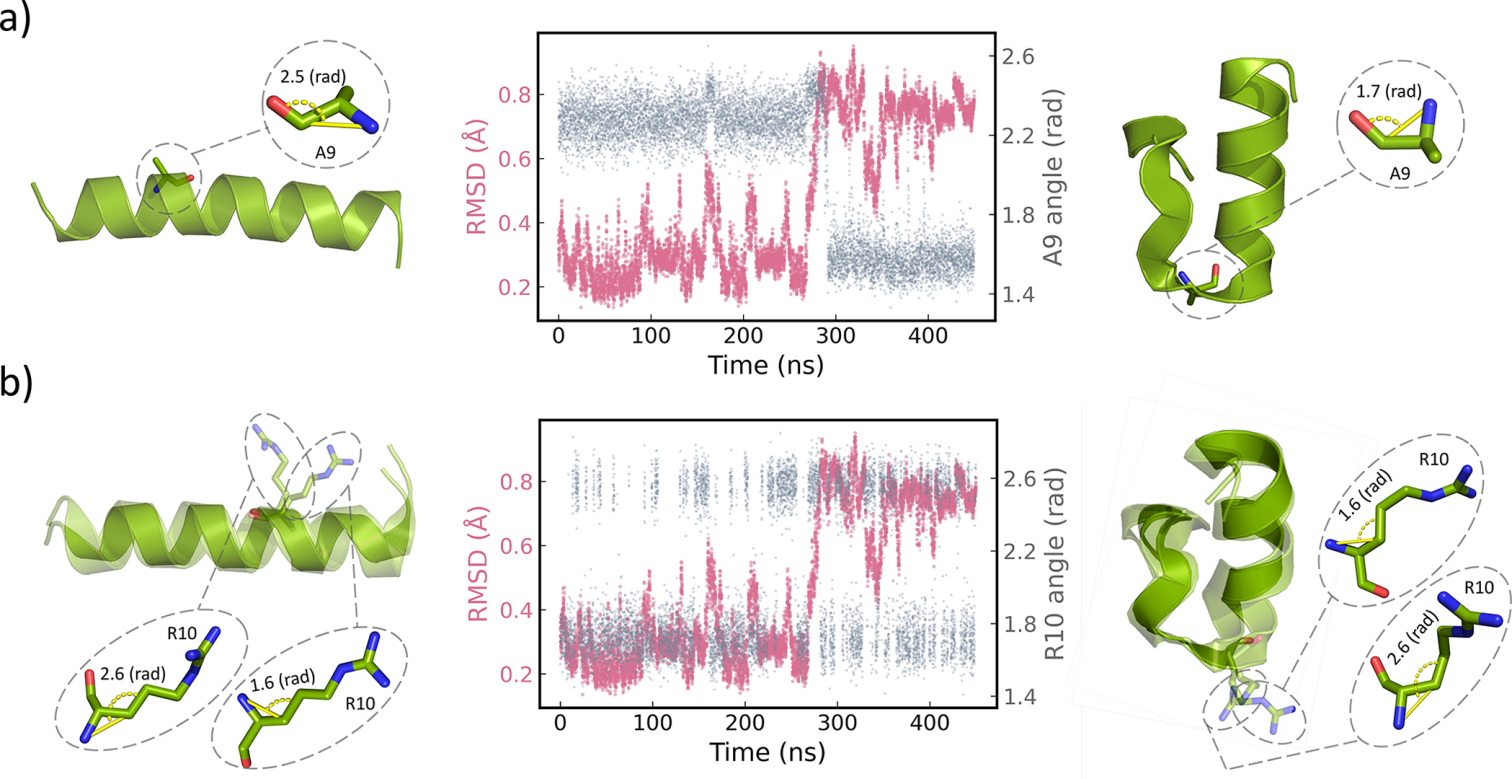
The A9 and R10 residues in Fs peptide protein are identified as SS and US residues, respectively. (a) The A9 residue (SS residue) exhibits a strong correlation with the RMSD. (b) The R10 residue (US residue) typically oscillates between the two angular states throughout the protein dynamics. The protein conformations are shown using PyMOL representation.^58^

**Figure 5:**
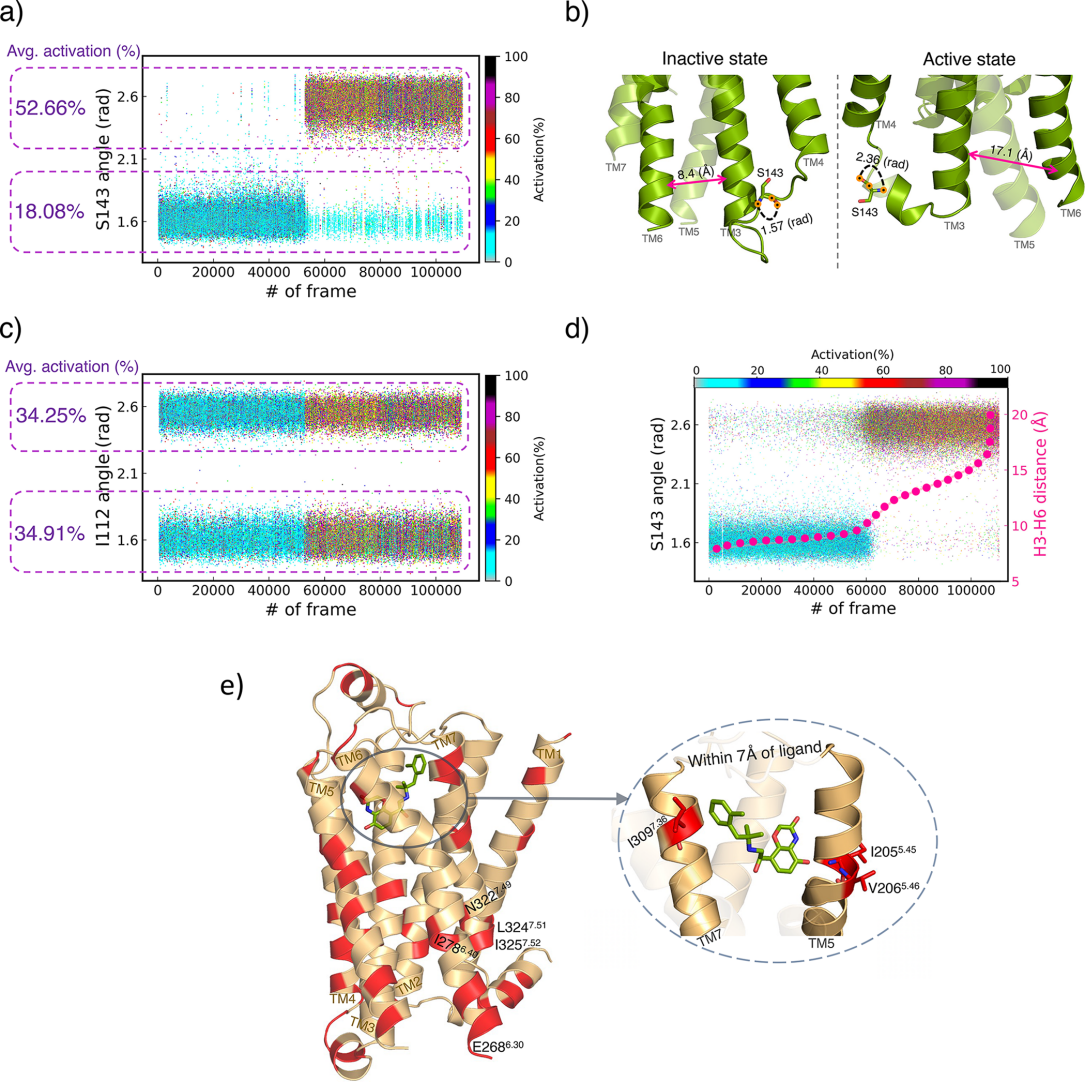
The *S*143^34.55^ and *I*112^3.31^ residues in β_2_*AR* receptor classified as SS and US residues, respectively. (a) The *S*143^34.55^ residue represents a strong correlation to activation states of the receptor. (b) Conformational representation^58^ of the receptor in the active and inactive states show the correlation between H3–H6 distance (measured as *C*_α_ contact distance between *R*131^3.50^–*L*272^6.34^ residues) and angular states in the *S*143^34.55^ residue. (c) The *I*112^3.31^ residue fluctuates between the two angular states throughout the protein dynamics. (d) The switching function of *S*143^34.55^ residue is correlated to the activation states and H3–H6 distances in the receptor. (e) All switch residues over β_2_*AR* receptor structure are highlighted in red (see Table S2 for a list of switch residues in this receptor). The *I*205^5.45^, *V*206^5.46^, and *I*309^7.36^ switch residues are within 7 Å of the ligand.

The global properties of proteins describe characteristics as a whole rather than focusing on specific local regions or individual amino acids. Some common examples of global features of proteins include their stability, activity, folding patterns, and functional properties. We observed that the switchlike transition between the two angular states in SS residues occurs when there is a notable change in the overall properties of the protein. On the other hand, there is no significant correlation between the US residue dynamics and essential changes in the global features of proteins. In order to validate our findings, we evaluated the SS and US residues within the Fs peptide protein as well as in the β_2_*AR* receptor. We examined the global characteristics of these proteins that were associated with SS residues (shown in Figure 4 and Figure 5). It is essential to emphasize that compared to traditional methods such as correlation analysis, the method we developed works with density of angular states (histograms), which incorporates the statistical information derived from thousands of trajectories in the case of heavy proteins. However, the correlation coefficient methods are not based on the collective behavior of atoms. When it comes to analyzing complex, multidimensional relationships, the use of ML techniques, like the methods we have developed, becomes imperative. Another advantage of our method compared to traditional methods is its computational efficiency. We first filter out the SS residues and then work only on them to specify their correlation to protein properties. We postulate that in the case of traditional methods, there is no means to have such prior knowledge, which results in redundant calculations.

### SS Residue in Fs Peptide Protein Correlated to RMSD

In this study, we used trajectories of the Fs peptide protein (Ace-A_5(AAARA)_3A-NME), which is a well-established model system for studying protein folding. The trajectory is 500 ns in duration and is saved every 50 ps. The simulation was executed using OpenMM 6.0.1 and the AMBER99SB-ILDN force field with GBSA-OBC implicit solvent at 300 K.^57^ It was initiated from randomly selected conformations obtained from an initial 400 K unfolding simulation.^57^ For this trajectory, the RF model classified seven residues (A3, A7, A9, R10, R15, R20, and A22) as the switch residues out of 21 residues within the protein (Table 2). The A9 residue is also detected as the SS residue. In Table 2, the angle switch ratio (ANSR) is defined as the ratio of switch angles over the total angles in a residue. For example, 10 angles can be formed within the A9 residue, and only two of them exhibit switching functions. Thus, the ANSR for the A9 residue will be 2/10. It is reasonably assumed that residues with higher ANSR values would have stronger correlations with global properties of a protein. The atom switch contribution (ATSC) highlights the degree of contribution of each atom within a residue to its switching function. For example, the two switch angles in residue A9 are formed between the O–C–N and the O–CA–N atoms. Hence, the ATSC for atoms O, C, CA, and N will be 2, 1, 1, and 2, respectively. After identifying the SS residue in the Fs peptide protein, we proceeded to assess any correlation with the global properties of the protein. In this study, we specifically focused on the folding process of the protein, as indicated by the Root Mean Square Deviation (RMSD) feature. As shown in Figure 4a, there is a strong correlation between the SS residue and the RMSD feature in the protein. At time ∼280 ns, the RMSD significantly increases; simultaneously, the A9 residue (SS residue) switches to another angular state. On the other hand, the R10 residue (US residue) mostly oscillates between the two states throughout the folding process, regardless of the significant change in the RMSD.

**Table 2:** List of Amino Acid Residues in Fs Peptide Protein Classified as Switch Residues Using the RF Model^a^

Residue	ANSR	ATSC	Residue	ANSR	ATSC
**ALA9**	2/10	O:2, N:2, C:1, CA:1	ARG10	6/120	CG:5, CB:4, C:3, N:2, NE:2, CA:1, CD:1
ALA7	1/10	O:1, C:1, N:1	ARG15	3/120	CG:3, C:2, CB:2, CA:1, NE:1
ALA3	1/10	O:1, CA:1, N:1	ARG20	1/120	CB:1, CD:1, NE:1
ALA22	1/10	O:1, C:1, N:1			

a ALA9 is detected as SS residue. The angle switch ratio (ANSR) and atom switch contribution (ATSC) are represented for the switch residues.

### SS Residues in β_2_*AR* Receptor Correlated to Activation States

In some proteins, certain global properties such as protein-drug binding, protein folding, and major conformational changes necessary for protein function may occur within microseconds to millisecond,^59^ which corresponds to the time required for SS residues to jump between the angular states. Running such long MD simulations is computationally expensive when dealing with heavy proteins. In such scenarios, instead of relying on long trajectories, employing thousands of short trajectories can assist us in capturing the switching functions of amino acids. For this case, we worked on G protein-coupled receptors (GPCRs) that are membrane proteins and play crucial roles in transmitting signals into cells. When a ligand binds to the GPCR, it induces conformational changes in the receptor, causing the receptor to be activated. The activation states are essential for receptor functions and associated with physiological responses. The study of switch residues in GPCRs can shed light on the mechanisms of cell signaling and the transition pathways between activation states in the receptors at residue level.^1,28,60−63^ We analyzed the conformational changes in β_2_*AR* receptor which plays a crucial role in regulating bronchodilation, heart rate, and blood pressure.^64−66^ We assessed the switch residues and the correlation between the SS residues and activation states in this receptor. For this study, we randomly selected 5000 MD short trajectories (consisting of 110,000 frames) representing structures of β_2_*AR* receptor in the presence of the partial inverse agonist carazolol. The simulations were performed for both the inactive and active crystal structures of the receptor (referred to as PDB 2RH1(67) and PDB 3P0G,^53^ respectively). The protein was embedded in a POPC lipid bilayer and solvated with water. The TIP3P water model was used in this simulation. The simulation snapshots were saved every 0.5 ns. All simulations were carried out with the Gromacs 4.5.3 MD package on Google Exacycle.^53,68^

Table 3 shows a list of SS residues in the β_2_*AR* receptor (see Table S2 for list of US residues). Similar to Table 2, the ANSR and ATSC metrics are reported for the switch residues. The *E*268^6.30^ and *S*143^34.55^ residues displayed US and SS functions, respectively. During the inactive states of the receptor, the *S*143^34.55^ residue interacts with *D*130^3.49^ residue within the conserved DRY motif (*D*130^3.49^, *R*131^3.50^, *Y* 132^3.51^).^69,70^ The DRY region forms an energy barrier that must be broken to achieve the activated state that is necessary for G protein coupling and downstream cellular responses.^71,72^ The DRY motif is also involved with highly conserved *E*268^6.30^ switch residue to form an “ionic lock” associated with the receptor’s inactive state.^72−74^ These interactions are essential for stabilizing conformations and the function of the receptor. For GPCR’s overall property, we focused on the activation states of the receptor.

**Table 3:** List of SS Residues in the β_2_*AR* Receptor^a^

Residue	ANSR	ATSC	Residue	ANSR	ATSC
*S*143^34.55^	6/20	OG:4, N:4, C:3, CA:3, CB:2, O:2	*Q*142^34.54^	5/84	C:3, CG:3, N:3, CA:2, CB:2, O:2
*I*121^3.40^	12/56	CG1:7, CG2:6, CA:5, CD1:5, CB:4, N:4, C:3, O:2	*Y* 219^5.58^	2/35	N:2, OH:2, C:1, CA:1
*I*278^6.40^	4/56	CG2:3, C:2, CA:2, CB:2, N:1, CG1:1, CD1:1	*M*215^5.54^	3/56	CG:3, C:2, CB:2, CA:1, N:1

a See Table S2 for the list of US residues. The angle switch ratio (ANSR) and atom switch contribution (ATSC) are reported for the switch residues. The Ballesteros–Weinstein numbers are utilized to represent the amino acids.

In our previous work,^62^ we introduced an XGBoost model that predict the activation state (classification task) and activity level (regression task) of a given receptor with prediction accuracy of 97.27% and 8.55% MAE, respectively. We applied that model to the data set employed in this study with the aim of estimating the correlation between the activation states and dynamics of SS residues in the receptor. Figure 5a demonstrates how this correlation appeared in the *S*143^34.55^ residue. As the angle in the residue is in the range of ∼1.5–1.8 (rad), the average activity level of the receptor is 18.08% and it increases to 52.66% for angles in ∼2.4–2.7 (rad). Figure 5b displays conformational representations of the angles in the inactive and active structures of the receptor. As shown in Figure 5c, the *I*112^3.31^ residue (US residue) mostly fluctuates between the two angular states during the protein dynamics. The Figure shows that the average activation states of the receptor for both angular states are 34.25% and 34.91%, meaning that the dynamic of the *I*112^3.31^ residue is not correlated to the activation states of the entire protein. To verify the strength of our models, we incorporated a critical structural feature of the receptor into this analysis. Helix3 (H3) and helix6 (H6) are two of the seven transmembrane helices that make up the core structure of GPCRs. Changes in the H3–H6 distance have essentially significant contributions to downstream signaling pathways and activation states of the receptor. In this study, we measured the H3–H6 distance as the *C*_α_ contact distance between *R*131^3.50^–*L*272^6.34^ amino acids. Figure 5d illustrates that the dynamics of *S*143^34.55^ as the SS residue, activation states of the receptor, and H3–H6 distances are heavily correlated to each other. The other SS residues shown in Table 3 (*I*121^3.40^, *I*278^6.40^, *Q*142^34.54^, *Y* 219^5.58^, and *M*215^5.54^) represent similar correlations to the activation states and H3–H6 distances. Figure 5e highlights all switch residues throughout the β_2_*AR* receptor structure in red. The *N*322^7.49^, *L*324^7.51^, and *I*325^7.52^ switch residues belong to the NPLIY motif within the receptor. The NPxxY motif is a conserved sequence in GPCRs (xx amino acids can vary in different GPCRs) that have significant effects on the activation process, impacting ligand affinity and G protein coupling.^75,76^ Furthermore, during the activation process of receptors, the conserved polar network in GPCRs undergoes essential rearrangements that contribute to stabilizing the receptor’s structure and aiding intracellular signaling.^77^ Our model detected the *I*278^6.40^ as SS residue within the polar network.^77^Figure 5e also shows that the *I*205^5.45^, *V* 206^5.46^, and *I*309^7.36^ switch residues are identified within 7 Å of the ligand. Understanding the dynamics and functions of amino acids within the binding pocket and its proximity can provide insights into ligand–protein interactions. Each of these switch residues can play significant roles in ligand-binding mechanisms and perform an induced fit for optimal signal transduction. Overall, our model demonstrates that those amino acids that are already known as crucial in the protein properties may exhibit switching function. Studying the dynamics of SS residues and their correlation with activation states in GPCRs provide valuable insights into the mechanisms of conformational changes in the receptors. This understanding will also facilitate the design of more effective drugs targeting GPCRs by specifically interacting with the key amino acids associated with activation states in the receptors.

## Conclusion

In this study, we developed a framework to characterize the structure and dynamics of amino acid residues in proteins using MD simulations and ML models. We mainly focused on residues containing angle(s) that switch between two distinct angular states without passing through any intermediate states. We implemented ML models to classify such residues in proteins. Our analysis indicated that the Random Forest model achieved an accuracy of 96.94% in classifying switch residues. Notably, our study revealed that switch residues, despite exhibiting similar densities of angular states, can differ substantially in terms of their structural dynamics and stability. The stable switch (SS) residue tends to remain stable in one of the angular states, and its conformation changes with a single transition to another state during the protein dynamics, while the unstable switch (US) residue constantly fluctuates between the two angular states. To distinguish between SS and US residues, we developed another method using the Logistic Regression model. This model exhibits an accuracy of 98.97% in classifying these residues. In addition, we found that there is a strong correlation between the dynamics of SS residues and the protein’s global properties. We confirmed the validity of it by evaluating the correlation between SS residue and folding characteristics as RMSD in the Fs peptide protein. Additionally, this study has demonstrated the correlation between the SS residues and activation states in β_2_*AR* receptor. This knowledge of the switching function of amino acids serves as a foundation for advancing our understanding and analysis of protein characteristics, which can be applied to protein engineering, protein-based therapeutics, and drug discovery approaches.

## Data Availability

The necessary information containing the training data sets, codes, and scripts for ML models used in this study is available here: https://github.com/pmollaei/AminoSwitch.

## Supporting Information Available

The Supporting Information includes selected atoms in amino acids, a comparison between basic vs dihedral angles, the Instability ratio, and a list of switch residues in the β_2_*AR* receptor (PDF)
