# Supplementary material for: Unveiling Switching Function of Amino Acids in Proteins Using a Machine Learning Approach
Source: J Chem Theory Comput. 2023 Nov 7;19(22):8472–80. doi: 10.1021/acs.jctc.3c00665 (PMC10688191; doi:10.1021/acs.jctc.3c00665)
Supplement: Supplementary file 1 — ct3c00665_si_001.pdf [file ct3c00665_si_001.pdf]

Supporting Information: Unveiling Switching Function of Amino Acids in Proteins Using a Machine Learning Approach

Parisa Mollaei[†] and Amir Barati Farimani[*,†,‡,¶]

†*Department of Mechanical Engineering, Carnegie Mellon University, 5000 Forbes Ave, Pittsburgh, PA 15213, United States*

‡*Department of Biomedical Engineering, Carnegie Mellon University, 5000 Forbes Ave, Pittsburgh, PA 15213, United States*

¶*Machine Learning Department, Carnegie Mellon University, 5000 Forbes Ave, Pittsburgh, PA 15213, United States*

E-mail: barati@cmu.edu

1 Selected atoms in amino acids

To determine angles within individual amino acids, we considered them as rigid bodies and chose atoms positioned at their edges and nodes. These selected atoms effectively capture the overall structure and dynamics of the amino acids. Table S1 shows the list of chosen atoms within each amino acid.

2 Basic vs dihedral angles

In general, angles formed among three atoms describe local geometry around individual atoms with more details and achieve more information about the spatial arrangement of

Table S1. Selected atoms within amino acids structures.

Residue	Structure	Selected Atoms	Residue	Structure	Selected Atoms
ALA	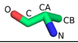	O, C, CA, CB, N	*GLN*	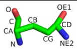	O, C, CA, N, CB, CG, CD, OE1, NE2
GLU	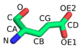	O, C, CA, N, CB, CG, OE2, CD, OE1	*LYS*	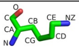	O, C, CA, N, CB, CG, CD, CE, NZ
GLY	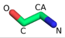	O, C, CA, N	*ILE*	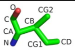	O, C, CA, N, CB, CG1, CG2, CD
THR	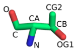	O, C, CA, N, CG2, CB, OG1	*TRP*	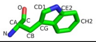	O, C, CA, N, CB, CG, CD1, CE2, CH2
PHE	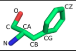	O, C, CA, N, CB, CG, CZ	*CYS*	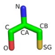	O, C, CA, N, CB, SG
SER	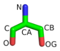	O, C, CA, N, CB, OG	*PRO*	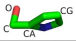	O, C, CA, CG
ASP	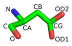	O, C, CA, N, CB, CG, OD1, OD2	*ASN*	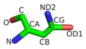	O, C, CA, N, CB, CG, ND2, OD1
VAL	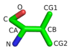	O, C, CA, N, CB, CG1, CG2	*MET*	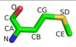	O, C, CA, N, CB, CG, SD, CE
TYR	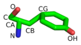	O, C, CA, N, CB, CG, OH	*HIS*	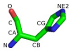	O, C, CA, N, CB, CG, NE2
LEU	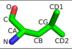	O, C, CA, N, CB, CG, CD1, CD2	*ARG*	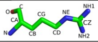	O, C, CA, N, CB, CG, CD, NE, CZ, NH1, NH2

atoms around a central atom and dynamics of an amino acid in comparison with those created among four atoms (dihedral angles). Importantly, as Table 2 in the main manuscript shows, seven amino acids in Fs peptide protein have been identified as switch residues using the basic angles (between sets of three atoms), however, only ALA9 amino acid exhibits switching function using dihedral angles. This confirms that some structural information is overlooked when relying only on dihedral angles (see GitHub repository for scripts of both basic and dihedral angle analyses). Fig.S1 displays plots of basic (a) and dihedral (b) angles within ALA22 amino acid in the Fs peptide protein. Although the amino acid is classified as the switch residue using the basic angles (the switch angle is highlighted in red in Fig.S1a), it is identified as non-switch residue based on dihedral angles (Fig.S1b). It is important to clarify that all angular states with single minima may not satisfy the switch definition. It is obvious that the switch residue is defined as amino acids switching between two distinct angular states with no intermediate state, as only the highlighted angular state in Fig.S1a meets this definition.

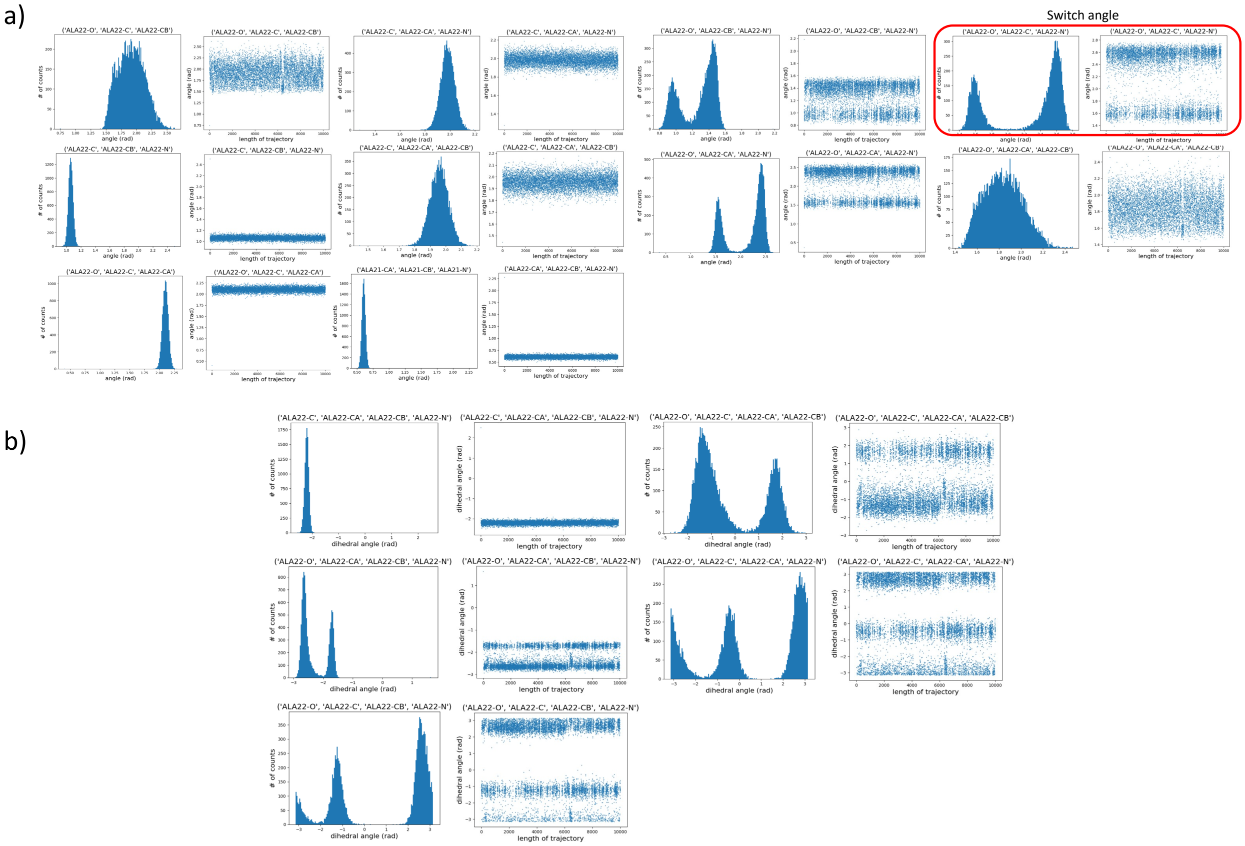

Fig.S1. Basic (a) vs dihedral (b) angles within ALA22 amino acid in Fs peptide protein. The identified switch angle formed by basic angles is highlighted in red. The plot titles represent the residue-atom that formed the angles.

3 Instability ratio

Our study uncovered that switch residues, despite having similar densities of angular states, can significantly vary in terms of their structural dynamics and stability. Specifically, the stable switch (SS) residue remains stable in one angular state, undergoing a single transition to another state during protein dynamics. On the other hand, the unstable switch (US) residue constantly fluctuates between the two angular states. To detect them using ML models, we defined the Instability ratio:

$$\text{Instability ratio} = \frac{\text{total number of transitions between the two angular states}}{\text{length of trajectories}}$$

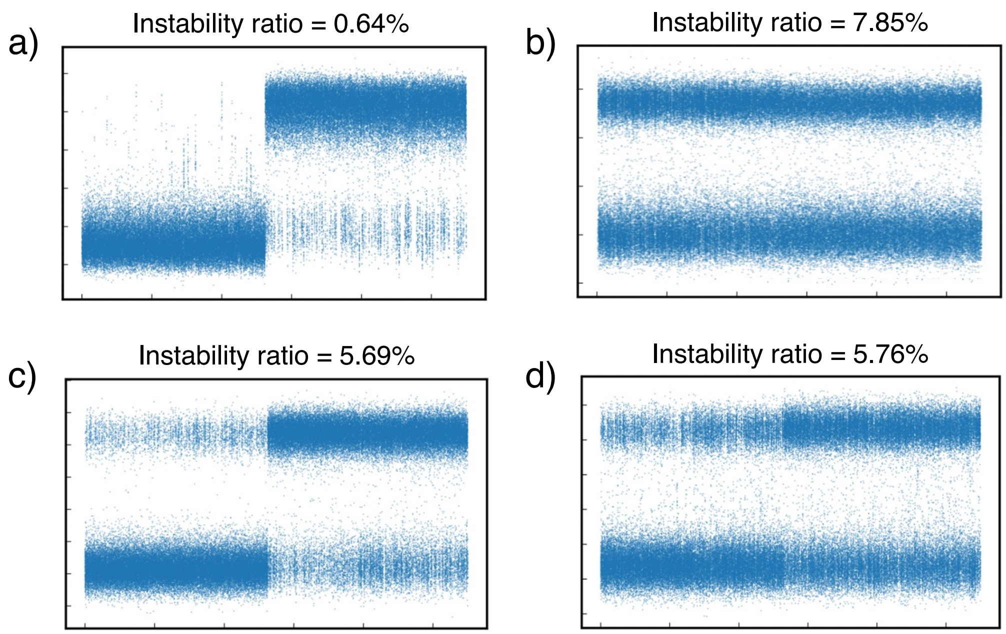

Fig.S2. Instability ratio as a metric to distinguish the SS and US residues. (a) Instability ratio <1% introduce SS residues, (b) Instability ratio >6% describe US residues. (c)(d) the stability of the states is significantly influenced by the distributions of transitions' timesteps for intermediate range of instability ratios (between 1% and 6%). Consequently, the Logistic Regression model is utilized to classify the SS and US residues.

In order to determine the total number of transitions between the two angular states, we employed k-means algorithm, which is an unsupervised clustering technique, implementing the Scikit-learn library. By identifying the state of each point along the trajectory, we were able to quantify the number of transitions occurring between the two states and subsequently calculate the Instability ratio. The Instability ratio effectively distinguishes the SS and US residues when it is either below 1% (Fig.S2a) or above 6% (Fig.S2b). However, differentiating between the two becomes difficult when the Instability ratio falls within the intermediate range (1%<Instability ratio<6%). This difficulty arises due to the varied distributions of transitions' timesteps between the two angular states throughout the trajectories. Some cases with very close Instability ratios in the intermediate range (e.g., 5.69% and 5.76%) can exhibit entirely different transition distributions over the trajectories, as depicted in Fig.S2c,d. To address this challenge, we used the Logistic Regression model to establish the

Table S2. List of residues in $\beta_2 AR$ receptor classified as bimodal switch residues using the RF model. The angle switch ratio (ANSR) and atom switch contribution (ATSC) are reported for the residues. Highlighted residues display the SS residues. The Ballesteros-Weinstein numbers are utilized to represent the amino acids.

Residue	ANSR	ATSC	Residue	ANSR	ATSC
$Y209^{5.48}$	12/35	CG:6, CB:6, OH:6, N:6, C:5, O:4, CA:3	$M279^{6.41}$	3/56	CG:3 , CB:2 , N:2 , C:1 , CA:1
$S143^{34.55}$	6/20	OG:4, N:4, C:3, CA:3, CB:2, O:2	$L53^{1.52}$	3/56	CA:3 , CD2:2 , CD1:2 , CB:1 , CG:1
$I121^{3.40}$	12/56	CG1:7, CG2:6, CA:5, CD1:5, CB:4, N:4, C:3, O:2	$L84^{2.55}$	3/56	CG:3 , N:2 , C:1 , CA:1 , O:1 , CB:1
$S74^{2.45}$	4/20	OG:4, C:2, CA:2, CB:2, N:2	$S220^{5.59}$	1/20	C:1 , CB:1 , OG:1
$S137^{3.56}$	3/20	OG:3, C:2, CB:2, CA:1, N:1	$S329^{8.47}$	1/20	C:1 , CA:1 , OG:1
$V152^{4.44}$	5/35	CG2:5, C:2, CA:2, CB:2, N:2, O:1, CG1:1	$K149^{4.41}$	4/84	CG:4 , C:3 , CB:2 , N:2 , CA:1
$V307^{7.34}$	4/35	CG2:4, C:2, CA:2, CB:2, N:2	$K140^{34.52}$	3/84	CG:3 , C:2 , CB:2 , CA:1 , N:1
$H269^{6.31}$	4/35	CG:4, C:2, CA:2, CB:2, N:2	$K147^{4.39}$	3/84	CG:2 , CB:2 , CE:2 , CA:1 , N:1 , CD:1
$L42^{1.41}$	6/56	CG:5, C:3, CA:3, CB:3, N:2, CD2:2	$I294^{6.56}$	2/56	CA:2 , CD1:2 , CB:1 , CG1:1
$I334^{8.52}$	6/56	CA:4, CG1:3, CB:3, C:2, CD1:2, N:2, CG2:2	$Q197^{5.37}$	3/84	CG:3 , C:2 , CB:2 , CA:1 , N:1
$M36^{1.35}$	5/56	CG:4, CA:3, CB:3, C:2, SD:2, N:1	$I325^{7.52}$	2/56	C:2 , CG1:2 , CA:1 , CB:1
$L144^{34.56}$	5/56	CG:4 , CA:3 , CB:3 , C:2 , CD2:2 , N:1	$I72^{2.43}$	2/56	CA:2 , CD1:2 , CB:1 , CG1:1
$L272^{6.34}$	5/56	CG:4 , CA:3 , CB:3 , C:2 , CD2:2 , N:1	$V206^{5.46}$	1/35	O:1 , CG1:1 , CG2:1
$V213^{5.52}$	3/35	CG2:3 , C:1 , CB:1 , CA:1 , N:1 , O:1 , CG1:1	$Q299^{6.61}$	2/84	C:2 , CG:2 , CA:1 , CB:1
$L145^{34.57}$	4/56	CB:3 , CG:3 , CA:2 , CD2:2 , C:1 , N:1	$E188^{-}$	2/84	C:2 , CG:2 , CA:1 , CB:1
$I182^{-}$	4/56	CA:3 , CG1:3 , C:2 , CB:2 , CD1:2	$K267^{6.29}$	2/84	CB:2 , CG:2 , CE:1 , N:1
$L324^{7.51}$	4/56	CA:3 , CG:3 , C:2 , CB:2 , CD2:2	$E268^{6.30}$	2/84	C:2 , CG:2 , CA:1 , CB:1
$I278^{6.40}$	4/56	CG2:3 , C:2 , CA:2 , CB:2 , N:1 , CG1:1 , CD1:1	$N322^{7.49}$	1/56	C:1 , CA:1 , OD1:1
$E338^{8.56}$	6/84	CG:5 , CA:4 , CB:3 , C:2 , CD:2 , N:1 , O:1	$L163^{4.55}$	1/56	C:1 , N:1 , CG:1
$Q142^{34.54}$	5/84	C:3 , CG:3 , N:3 , CA:2 , CB:2 , O:2	$I314^{7.41}$	1/56	CA:1 , CG1:1 , CD1:1
$K273^{6.35}$	5/84	CG:4 , C:3 , CB:3 , N:2 , CA:1 , CD:1 , CE:1	$I205^{5.45}$	1/56	C:1 , CG1:1 , CD1:1
$E180^{000}$	5/84	CG:4 , CA:3 , CB:3 , C:2 , CD:2 , N:1	$I298^{6.60}$	1/56	CA:1 , CG1:1 , CD1:1
$Q224^{5.63}$	5/84	CG:5 , C:3 , N:3 , CA:2 , CB:2	$I309^{7.36}$	1/56	CA:1 , CG1:1 , CD1:1
$Y219^{5.58}$	2/35	N:2 , OH:2 , C:1 , CA:1	$I153^{4.45}$	1/56	CA:1 , CG1:1 , CD1:1
$V31^{1.30}$	2/35	N:2 , CA:1 , CG1:1 , CB:1 , CG2:1	$R333^{8.51}$	2/120	CB:2 , NE:2 , CD:1 , CG:1
$V67^{2.38}$	2/35	N:2 , CA:1 , CG1:1 , CB:1 , CG2:1	$K60^{1.59}$	1/84	N:1 , CB:1 , CG:1
$D331^{8.49}$	3/56	CG:3 , C:2 , CB:2 , CA:1 , N:1	$Q179^{-}$	1/84	CA:1 , CB:1 , CD:1
$M215^{5.54}$	3/56	CG:3 , C:2 , CB:2 , CA:1 , N:1	$K270^{6.32}$	1/84	CA:1 , N:1 , CG:1
$M156^{4.48}$	3/56	CA:3 , SD:2 , CG:2 , CB:1 , N:1	$R151^{4.43}$	1/120	C:1 , N:1 , CG:1
$I112^{3.31}$	3/56	CG2:3 , C:2 , CA:2 , CB:1 , N:1			

Instability ratio for classifying the SS and US residues.

4 US and SS residues in $\beta_2 AR$ receptor

Table S2 shows the list of residues classified as US and SS in $\beta_2 AR$ receptor using the RF model. The table also presents the angle switch ratio (ANSR) and atom switch contribution (ATSC) for each switch residue. The ANSR represents the ratio of switch angles to the total angles within a single residue. The table is ordered based on the ANSR values. As shown in the table, the $Y209^{5.48}$ residue contains the most angle switch ratio within the $\beta_2 AR$ receptor. It is a justifiable assumption that residues with higher ANSR values demonstrate stronger correlations with the global properties of a protein. The ATSC quantifies the extent

of contribution of each atom within a residue to its switching function.
